# Phase Ib/II Study of a Liposomal Formulation of Eribulin (E7389-LF) plus Nivolumab in Patients with Advanced Solid Tumors: Results from Phase Ib

**DOI:** 10.1158/2767-9764.CRC-22-0401

**Published:** 2023-07-10

**Authors:** Hanae Ida, Toshio Shimizu, Makoto Nishino, Yoshiaki Nakamura, Shu Yazaki, Yuki Katsuya, Jun Sato, Takafumi Koyama, Satoru Iwasa, Kazuki Sudo, Shunsuke Kondo, Kan Yonemori, Kohei Shitara, Satoshi Shiono, Daiko Matsuoka, Keisuke Yasuda, Yohei Otake, Takuya Suzuki, Takao Takase, Shuya Takashima, Kohei Yamaguchi, Taro Semba, Noboru Yamamoto

**Affiliations:** 1 Department of Experimental Therapeutics, National Cancer Center Hospital, Tokyo, Japan.; 2 Department of Gastroenterology and Gastrointestinal Oncology, National Cancer Center Hospital East, Chiba, Japan.; 3 Department of Medical Oncology, National Cancer Center Hospital, Tokyo, Japan.; 4 Department of Gastrointestinal Medical Oncology, National Cancer Center Hospital, Tokyo, Japan.; 5 Department of Hepatobiliary and Pancreatic Oncology, National Cancer Center Hospital, Tokyo, Japan.; 6 Department of Immunology, Nagoya University Graduate School of Medicine, Nagoya, Japan.; 7 Oncology Early Clinical Operation II, Ono Pharmaceutical Co., Ltd., Osaka, Japan.; 8 Japan and Asia Clinical Development Department, Oncology Business Group, Eisai Co., Ltd., Tokyo, Japan.; 9 Clinical Data Science Department, Medicine Development Center, Eisai Co., Ltd., Tokyo, Japan.; 10 Clinical Pharmacology Science Department, Medicine Development Center, Eisai Co., Ltd., Tokyo, Japan.; 11 Oncology Tsukuba Research Department, Oncology Business Group, Eisai Co., Ltd., Ibaraki, Japan.; 12 Department of Thoracic Oncology, National Cancer Center Hospital, Tokyo, Japan.

## Abstract

**Purpose:**

To determine a recommended dose of liposomal eribulin (E7389-LF) in combination with nivolumab in patients with advanced solid tumors, and to evaluate the safety, efficacy, pharmacokinetics, and biomarker impact of this regimen.

**Experimental Design:**

Japanese patients with advanced, nonresectable, or recurrent solid tumors and no existing alternative standard/effective therapy (except nivolumab monotherapy) were assigned to either E7389-LF 1.7 mg/m^2^ plus nivolumab 360 mg every 3 weeks, E7389-LF 2.1 mg/m^2^ plus nivolumab 360 mg every 3 weeks, E7389-LF 1.1 mg/m^2^ plus nivolumab 240 mg every 2 weeks, or E7389-LF 1.4 mg/m^2^ plus nivolumab 240 mg every 2 weeks. Primary objectives were to evaluate the safety/tolerability of each dose cohort and to determine the recommended phase II dose (RP2D). Secondary/exploratory objectives, including safety [dose-limiting toxicities (DLT) and adverse events (AE)], pharmacokinetics, efficacy [including objective response rate (ORR)], and biomarker results were used in determining the RP2D.

**Results:**

Twenty-five patients were enrolled to treatment [E7389-LF 1.7 mg/mg^2^ every 3 weeks (*n* = 6), E7389-LF 2.1 mg/m^2^ every 3 weeks (*n* = 6), E7389-LF 1.1 mg/m^2^ every 2 weeks (*n* = 7), E7389-LF 1.4 mg/m^2^ every 2 weeks (*n* = 6)]. Twenty-four patients were evaluated for DLTs, of whom 3 had DLTs (1 at E7389-LF 1.7 mg/m^2^ every 3 weeks, 1 at 1.1 mg/m^2^ every 2 weeks, and 1 at 1.4 mg/m^2^ every 2 weeks). All patients had ≥1 treatment-related treatment-emergent AE (TEAE); 68.0% had ≥1 grade 3–4 treatment-related TEAE. Changes in vasculature and IFN-related biomarkers were seen in each cohort. The overall ORR was 16%.

**Conclusions:**

E7389-LF plus nivolumab was tolerable overall; the recommended dose for future study was 2.1 mg/m^2^ plus nivolumab 360 mg every 3 weeks.

**Significance:**

This phase Ib part of a phase Ib/II study assessed the tolerability and activity of a liposomal formulation of eribulin (E7389-LF) plus nivolumab in 25 patients with advanced solid tumors. The combination was tolerable overall; 4 patients had a partial response. Vasculature and immune-related biomarker levels increased, suggesting vascular remodeling.

## Introduction

Eribulin is a halichondrin-class microtubule dynamics inhibitor with cytotoxic and vascular remodeling effects leading to tumor immune modulation (1–3). It has demonstrated efficacy in the treatment of patients with locally recurrent/advanced or metastatic breast cancer, who had been previously treated with regimens including an anthracycline and a taxane (4), and patients with locally recurrent/advanced or metastatic liposarcoma or leiomyosarcoma (5).

Liposomal formulations can be used to improve the access of anticancer treatment into tumor cells by increasing their ability to permeate tumor vasculature (6, 7). A liposomal formulation of eribulin (E7389-LF) was designed to enhance antitumor activity and improve the pharmacokinetic profile of eribulin. Pharmacokinetic comparisons of data from a nonclinical study (8) and phase I studies in patients with solid tumors (9, 10) have shown that E7389-LF has a longer (but not necessarily higher) exposure than the nonliposomal formulation of eribulin. Importantly, no unexpected safety signals were found with E7389-LF compared with eribulin (9).

E7389-LF monotherapy has shown responses in several cancer types; specifically, in the dose-escalation part of a phase I study (Study 114) enrolling 21 patients with solid tumors in Japan (10), an objective response was achieved among patients with adenoid cystic carcinoma, esophageal cancer, urothelial cancer, and uterine small cell cancer, resulting in an objective response rate (ORR) of 19% [95% confidence interval (CI), 5.4–41.9]. The most common treatment-emergent adverse events (TEAEs) of grade ≥3 were neutropenia and leukopenia (10), and the recommended dose regimen of E7389-LF for dose expansion was determined to be 2.0 mg/m^2^ of free-base eribulin every 3 weeks. This regimen was evaluated in multiple dose-expansion cohorts of Study 114 (11, 12). When assessed in 28 patients with metastatic breast cancer, the ORR was 35.7% (95% CI, 18.6–55.9), and the most common treatment-related TEAEs of grade ≥3 in this study were also neutropenia and leukopenia (11). Among patients (*n* = 43) with advanced solid tumors (adenoid cystic carcinoma, gastric cancer, esophageal cancer, and small cell lung cancer), the ORR was 11.6% (95% CI, 3.9–25.1), and again, the most common treatment-related TEAEs of grade ≥3 were neutropenia and leukopenia (12).

Chemotherapies such as eribulin have potential to provide synergistic antitumor effects through the programmed cell death-1 (PD-1) pathway when combined with immune checkpoint inhibitors (ICI; refs. 13, 14). In preclinical models, eribulin has shown efficacy in combination with PD-1 inhibitors at higher dose intensities (3), possibly due to eribulin's effect on the tumor microenvironment, including vascular remodeling (2, 15). For this reason, the combination of a longer-lasting liposomal formulation of eribulin with an immunotherapy presents a promising anticancer strategy and is expected to show antitumor activity by cytotoxic and antitumor immune effects.

In this study, we sought to determine a recommended phase II dose (RP2D) of E7389-LF in combination with nivolumab (16) in patients with advanced solid tumors, and to determine the safety, efficacy, pharmacokinetics, and biomarker impact of this regimen.

## Materials and Methods

### Study Design

Study 120 was an open-label phase Ib/II study of E7389-LF plus nivolumab in patients with advanced, nonresectable, or recurrent solid tumors. The primary objectives of the phase Ib part of Study 120 were to determine the safety, tolerability, and the RP2D of E7389-LF plus nivolumab. Secondary objectives of the phase Ib part included assessment of the pharmacokinetics of E7389-LF plus nivolumab. The key exploratory objectives of the phase Ib part were to assess ORR and explore potential biomarkers. E7389-LF plus nivolumab was administered to four cohorts comprising two dosing schedules (either every 3 weeks, on day 1 of a 21-day cycle, or every 2 weeks, on days 1 and 15 of a 28-day cycle) and two different doses of study drugs per schedule, both administered intravenously. These cohorts were: (i) E7389-LF 1.7 mg/m^2^ plus nivolumab 360 mg every 3 weeks; (ii) E7389-LF 2.1 mg/m^2^ plus nivolumab 360 mg every 3 weeks; (iii) E7389-LF 1.1 mg/m^2^ plus nivolumab 240 mg every 2 weeks; (iv) E7389-LF 1.4 mg/m^2^ plus nivolumab 240 mg every 2 weeks. Henceforth, cohorts will be referred to by the corresponding dose and schedule of eribulin. Per a decision by the sponsor, the E7389-LF doses used in this study were calculated according to eribulin mesylate content; they were determined by the maximum tolerated dose (MTDs) of E7389-LF monotherapy in Study 114 (10). Specifically, doses of E7389-LF were reduced as compared with Study 114 purely as a precautionary measure to compensate for the addition of nivolumab; no specific overlapping toxicities were anticipated. As the MTDs of E7389-LF monotherapy in Study 114 were 2.0 mg/m^2^ every 3 weeks and 1.5 mg/m^2^ every 2 weeks of free-base eribulin (10), these would equate to 2.3 mg/m^2^ every 3 weeks and 1.7 mg/m^2^ every 2 weeks of eribulin mesylate.

The two lower doses of E7389-LF in our study (1.7 mg/m^2^ every 3 weeks and 1.1 mg/m^2^ every 2 weeks) were chosen to be one dosing level below the MTDs in Study 114 (10), while the two higher doses in our study were chosen as a percent reduction from the MTDs in Study 114 (2.1 mg/m^2^ every 3 weeks was a 10% reduction of 2.3 mg/m^2^ mesylate; 1.4 mg/m^2^ every 2 weeks was a 20% reduction of 1.7 mg/m^2^ mesylate). In our study, patient enrollment began sequentially with these lower doses, and then to the higher doses if the lower doses were tolerated. In addition, when accounting for the time between doses for each schedule (either 2 weeks or 3 weeks), the planned dose intensities of E7839-LF in our study were similar between the lower and higher every-2-weeks and every-3-weeks doses. For regimens i (1.7 mg/m^2^ every 3 weeks) and iii (1.1 mg/m^2^ every 2 weeks), the planned dose intensity was 0.55 mg/m^2^/week, and for regimens ii (2.1 mg/m^2^ every 3 weeks) and iv (1.4 mg/m^2^ every 2 weeks), it was 0.70 mg/m^2^/week.

### Patients

This phase Ib study included patients with advanced, nonresectable, or recurrent solid tumors for which no alternative standard therapy or no effective therapy existed (patients eligible to receive nivolumab monotherapy as a standard of care were eligible for inclusion). Patients were ≥20 years of age, with a life expectancy of ≥12 weeks, an Eastern Cooperative Oncology Group performance status (ECOG PS) of 0 or 1, and adequate organ function. Patients were not eligible if they were previously treated with eribulin (including E7389-LF) or: any anti-PD-1, anti-PD-ligand 1 (PD-L1), anti-PD-L2, anti-CD137, anti-CTLA-4 antibody; any other antibody or drug specifically targeting T-cell costimulation or checkpoint; or cancer vaccine therapy that resulted in a grade ≥3 immune-related adverse event (irAE) or any-grade irAE leading to treatment discontinuation. Patients were also required to have tumors accessible for biopsy and had to consent to biopsy before and after treatment with study drug. A full list of inclusion and exclusion criteria can be found in the Supplementary Materials and Methods. At least 6 patients were enrolled in each cohort to assess safety [including dose-limiting toxicities (DLT)], biomarkers, pharmacokinetic profiles, and efficacy for each dose regimen.

This study was conducted in accordance with standard operating procedures of the sponsor, which were based on the Principles of the World Medical Association Declaration of Helsinki, all applicable Japanese Good Clinical Practices and regulations, and the Pharmaceutical Affairs Law for studies conducted in Japan. Written informed consent was obtained from all participants and these and the study protocol were reviewed and approved by the applicable institutional ethical review board.

### Study Procedures and Assessments

The tolerability of each cohort was assessed mainly by the incidence of DLTs during cycle 1 of phase Ib. DLTs were treatment-related TEAEs/toxicities listed in Supplementary Table S1; dosing cohorts were considered “tolerable” if ≤1 DLT occurred. Safety assessments consisted of monitoring and recording all adverse events (AE), including all Common Terminology Criteria for Adverse Events (CTCAE) grades (for both increasing and decreasing severity), and serious AEs (SAE), as well as regular laboratory evaluations.

Blood samples for pharmacokinetic analyses of E7389-LF were collected predose, 5 minutes, 0.5, 1, 2, 4, 6, 8, 24, 72, and 168 hours after the end of the infusion on cycle 1 day 1 (cycle # day #; C#D#). Plasma concentrations of eribulin were measured by a validated LC/MS-MS method. For nivolumab, blood samples were collected predose and postdose (just before completion of infusion) on C1D1, predose on C2D1, C3D1, and C4D1, predose and postdose on C5D1, and predose on C9D1 and C13D1. For the every-2-weeks dosing schedule, predose samples on C1D15 and C5D15 were also taken. Serum concentrations of nivolumab were measured by validated electrochemiluminescence immunoassay. For the biomarker analyses, blood samples were collected as follows: for the every-2-weeks schedule, blood samples were collected predose on C1D1, C1D8, C1D15, C1D22, each day 1 of cycle 2 and subsequent cycles (until cycle 7) and off-treatment. For the every-3-weeks schedule, samples were collected predose on C1D1, C1D8, C1D15, each day 1 of cycle 2 and subsequent cycles (until cycle 9) and off-treatment. Biomarkers were investigated with Angiogenesis MAP, Multiplex, and Simoa systems.

For efficacy analyses, tumor assessments were conducted by the investigator using RECIST version 1.1 (RECIST v1.1) every 6 weeks, at the off-treatment visit, and if clinically indicated.

Tumor biopsies were conducted at screening and on C2D1. Samples were used to investigate PD-L1 expression [by an approved IHC 28-8 pharmDx assay (Agilent Dako, Agilent Technologies)], assess PD-L1 combined positive score (CPS; %) based on PD-L1 expression in viable tumor cells and immune cells and categorize immune phenotypes (by panCK/CD8 IHC analysis). For the determination of immune phenotype, panCK-CD8 was scored by a pathologist according to the density proportion score (DPS). The DPS creates a distribution of inflammatory cell densities within a tumor ranging from areas with few or no inflammatory cells to regions densely populated with inflammatory cells. The DPSs for stained immune cells were assessed in two compartments: tumor stroma and tumor epithelial nests. The definition and terminology of phenotype classification were modified from those used in the ABACUS (17) and IMvigor210 (18) trials: a patient's phenotype was considered “immune-inflamed” if ≥20% of tumor epithelial nests had a moderate to high density of infiltrating CD8 cells. Patients with a lower DPS in tumor epithelial nests were categorized as “immune-excluded” if ≥20% of the tumor stroma had a moderate to high density of infiltrating CD8 cells. Cases were considered “immune-desert” if both tumor epithelial nests and tumor stroma had no, or only low, densities of infiltrating CD8 cells.

### Statistical Analyses

The RP2D was determined on the basis of the results of the DLT, safety, pharmacokinetic, efficacy, and biomarker analyses. AEs were listed according to Medical Dictionary for Regulatory activities version 24.1 and graded according to CTCAE version 5.0. The DLT evaluable set included all patients in phase Ib who received study drugs as planned in cycle 1 and patients who had DLTs. The safety analysis set included all patients who received at least one dose of E7389-LF or nivolumab. Pharmacokinetic analyses were performed on the pharmacokinetic analysis set (all patients who received at least one dose of E7389-LF or nivolumab and had at least one evaluable concentration datapoint). Noncompartmental pharmacokinetic parameters calculated for eribulin in phase Ib included the maximum plasma concentration of eribulin (C_max_), time at which the maximum plasma concentration occurs (t_max_), area under the plasma concentration–time curve (AUC), terminal elimination phase half-life (t_1/2_), total clearance (CL), and volume of distribution at steady state (V_ss_). The effect of E7389-LF plus nivolumab combination therapy on soluble, tissue, and/or genetic biomarkers was summarized using descriptive statistics.

ORR [proportion of patients with complete response (CR) or partial response (PR)] and disease control rate (DCR) [CR plus PR plus stable disease (SD) ≥5 weeks after starting treatment on C1D1] were assessed with 95% CI by cohort and in the overall population.

Pharmacodynamic changes of plasma biomarkers from baseline (or C1D1) were analyzed in biomarkers in which ≥80% of the samples demonstrated levels above the lower limit of quantification. Changes were assessed using the one-sample Wilcoxon signed-rank test. *P* values were adjusted using the Benjamini–Hochberg procedure for FDR control with the number of biomarkers analyzed at each timepoint. Statistical analyses and plots for biomarkers were performed and generated using Statistical Analysis Software v9.4 (SAS Institute Inc.). Changes in a focused group of biomarkers were investigated among the four dosing cohorts; these biomarkers included vasculature-related markers [collagen IV and TIE2 (tyrosine kinase immunoglobulin and EGF homology domains 2, also known as TEK receptor tyrosine kinase, TEK)] and IFN-related markers [IFNγ and IFNγ-induced protein 10 (IP10, also known as C-X-C motif chemokine ligand 10, CXCL10)].

These biomarkers were of specific interest, as E7389-LF was shown to increase the levels of these vasculature markers in a previous clinical trial (10), and the microvessel density of tumors in a preclinical study (19); in addition, E7389-LF (19) and nivolumab (20) have been shown to increase expression and induce signaling of IFNγ. PD-L1 CPS was calculated as the percentage of PD-L1-staining cells (including tumor cells, lymphocytes, and macrophages) over the total number of viable tumor cells. Finally, to investigate the effect on immune cells other than CD8 cells in patients with immune-phenotype change, gene expression profiling in biopsy tissues using an immune-cell-type gene set was conducted using nCounter PanCancer Immune Profiling Panel (NanoString). The expressions of the 44 cell type marker genes were visualized by heat maps. Genes were annotated on the basis of the information provided by NanoString with some modifications. The *CD4* gene was added as “CD4 T cells,” and the category name of *FOXP3* was changed from “Treg” to “CD4 T cells.” Raw expression values for baseline samples and log_2_ values for fold changes from baseline to C2D1 were used. On both of the heat maps, each gene was normalized by z-score normalization and patients were clustered with Ward method based on Euclidean distances.

### Data Availability Statement

Eisai Inc. commits to sharing data from clinical trials upon request from qualified scientific and medical researchers. Data requests are reviewed and authorized by an independent review panel on the basis of scientific merit, and data are anonymized with respect to applicable laws and regulations. Trial data availability is according to the criteria and process described on www.clinicalstudydatarequest.com.

## Results

### Patient Disposition and Baseline Characteristics

From September 5, 2019, to the data cut-off date (April 3, 2021), a total of 25 Japanese patients were enrolled and treated: 6 patients in the E7389-LF 1.7 mg/mg^2^ every-3-weeks cohort, 6 patients in the E7389-LF 2.1 mg/m^2^ every-3-weeks cohort, 7 patients in the E7389-LF 1.1 mg/m^2^ every-2-weeks cohort, and 6 patients in the E7389-LF 1.4 mg/m^2^ every-2-weeks cohort. At the data cut-off date, 2 patients (8.0%) were still undergoing treatment. The remaining 23 patients (92.0%) discontinued treatment: 19 patients (76.0%) due to disease progression, 3 patients (12.0%) due to patient choice, and 1 patient (4.0%) due to an adverse event. Baseline characteristics of patients are shown in Table 1. Of the 25 enrolled patients, the median age was 55 years (range, 34–79) and 16 (64.0%) were male. The majority of enrolled patients (88.0%) had an ECOG PS of 0. A total of 13 patients (52.0%) and 12 patients (48.0%) had received one to three lines and at least four lines of prior chemotherapy (respectively) for advanced or metastatic diseases; this distribution was similar to the total number of prior chemotherapy regimens received. In addition, 7 patients received prior perioperative chemotherapy, including 2 in the E7389-LF 1.1 mg/m^2^ every-2-weeks cohort (28.6%), 1 in the E7389-LF 1.4 mg/m^2^ every-2-weeks cohort (16.7%), 3 patients in the E7389-LF 1.7 mg/m^2^ every-3-weeks cohort (50.0%), and 1 in the E7389-LF 2.1 mg/m^2^ every-3-weeks cohort (16.7%). The representativeness of patients in this study as compared with real-world populations is available in Supplementary Table S2.

**TABLE 1:** Baseline patient characteristics

	Every 3 weeks^a^		Every 2 weeks^b^		
Characteristic	E7389-LF 1.7 mg/m^2^ *n* = 6	E7389-LF 2.1 mg/m^2^ *n* = 6	E7389-LF 1.1 mg/m^2^ *n* = 7	E7389-LF 1.4 mg/m^2^ *n* = 6	Total *N* = 25
**Median age, years (range)**	49.0 (42–66)	51.5 (34–79)	61.0 (50–70)	60.0 (44–69)	55.0 (34–79)
**Sex, *n* (%)**					
Male	3 (50.0)	4 (66.7)	5 (71.4)	4 (66.7)	16 (64.0)
Female	3 (50.0)	2 (33.3)	2 (28.6)	2 (33.3)	9 (36.0)
**ECOG PS, *n* (%)**					
0	5 (83.3)	6 (100)	5 (71.4)	6 (100)	22 (88.0)
1	1 (16.7)	0	2 (28.6)	0	3 (12.0)
**Median weight, kg (range)**	62.15 (43.9–76.6)	65.20 (29.8–109.4)	62.10 (50.4–77.5)	69.75 (57.2–85.7)	63.90 (29.8–109.4)
**Median BSA, m^2^ (range)**	1.661 (1.36–1.99)	1.765 (1.07–2.25)	1.650 (1.51–1.86)	1.814 (1.61–1.96)	1.738 (1.07–2.25)
**Tumor type, *n* (%)**					
Ovarian cancer	2 (33.3)	1 (16.7)	0	1 (16.7)	4 (16.0)
Thymic carcinoma	2 (33.3)	1 (16.7)	0	1 (16.7)	4 (16.0)
Gastric cancer	0	1 (16.7)	2 (28.6)	0	3 (12.0)
Cholangiocarcinoma	0	0	1 (14.3)	1 (16.7)	2 (8.0)
Colorectal cancer	1 (16.7)	0	1 (14.3)	0	2 (8.0)
Neuroendocrine carcinoma	0	1 (16.7)	0	1 (16.7)	2 (8.0)
Small cell lung cancer	0	1 (16.7)	1 (14.3)	0	2 (8.0)
Adenoid cystic carcinoma	1 (16.7)	0	0	0	1 (4.0)
Paget disease	0	0	1 (14.3)	0	1 (4.0)
Pancreatic cancer	0	0	0	1 (16.7)	1 (4.0)
Sarcoma	0	0	1 (14.3)	0	1 (4.0)
Primary origin unknown	0	1 (16.7)	0	0	1 (4.0)
Urothelial cancer	0	0	0	1 (16.7)	1 (4.0)
**Number of prior advanced or metastatic chemotherapy regimens, *n* (%)**					
1	1 (16.7)	1 (16.7)	0	0	2 (8.0)
2	1 (16.7)	2 (33.3)	2 (28.6)	0	5 (20.0)
3	0	0	5 (71.4)	1 (16.7)	6 (24.0)
≥4	4 (66.7)	3 (50.0)	0	5 (83.3)	12 (48.0)

Abbreviations: BSA, body surface area; E7389-LF, eribulin liposomal formulation; ECOG PS, Eastern Cooperative Oncology Group performance status.

^a^E7389-LF dose plus nivolumab 360 mg.

^b^E7389-LF dose plus nivolumab 240 mg.

### DLTs

One patient with colorectal cancer in the E7389-LF 1.1 mg/m^2^ every-2-weeks cohort was not included in the DLT evaluation, as this patient showed disease progression during cycle 1 and did not receive a dose on day 15. Thus, DLTs were observed in 3 of the 24 patients who received study drug as planned in cycle 1 (Supplementary Table S3), and all DLTs were resolved: in the every-3-weeks dosing cohort, 1 patient (who received E7389-LF 1.7 mg/m^2^) had grade 3 febrile neutropenia; in the every-2-weeks dosing cohort, 1 patient (who received E7839-LF 1.1 mg/m^2^) had grade 3 neutropenia requiring interruption of their day 15 dose and 1 patient (who received E7389-LF 1.4 mg/m^2^) had grade 3 febrile neutropenia.

### Safety

All patients (100%) had at least one treatment-related TEAE, and 68.0% of patients had at least one grade 3–4 treatment-related TEAE. The most frequently observed treatment-related TEAEs were leukopenia (72.0%) and neutropenia (68.0%; Table 2). Overall, the neutrophil-count nadir was observed between days 8 and 15 for both dosing schedules (Supplementary Fig. S1). This decrease in neutrophils (after the first dose of E7389-LF) was observed regardless of the dosing schedule. The most common grade 3–4 severity treatment-related TEAEs were neutropenia (52.0%), leukopenia (36.0%), and lymphopenia (16.0%; Table 2). Other grade 3–4 severity treatment-related TEAEs were anemia, febrile neutropenia, lipase increased (8.0% each), diarrhea, and amylase increased (4.0% each); no treatment-related deaths were observed in any cohort (Table 2). In addition, 4 patients (1 per cohort) received PEGylated GCSF after cycle 1 for the prevention of febrile neutropenia. Seven patients had treatment-emergent SAEs, most commonly pneumonia (*n* = 2, 8.0%; Supplementary Table S3). A treatment-related SAE of enterocolitis was observed in 1 patient in the 1.7 mg/m^2^ every-3-weeks cohort.

**TABLE 2:** Treatment-related TEAEs in ≥10% of all patients and all grade 3–4 treatment-related TEAEs

	Every 3 weeks^a^				Every 2 weeks^b^					
	E7389-LF 1.7 mg/m^2^ *n* = 6		E7389-LF 2.1 mg/m^2^ *n* = 6		E7389-LF 1.1 mg/m^2^ *n* = 7		E7389-LF 1.4 mg/m^2^ *n* = 6		Total *N* = 25	
Parameter	Any grade	Grade 3–4	Any grade	Grade 3–4	Any grade	Grade 3–4	Any grade	Grade 3–4	Any grade	Grade 3–4^c^
**Patients with any treatment- related TEAEs, *n* (%)**	6 (100)	5 (83.3)	6 (100)	4 (66.7)	7 (100)	4 (57.1)	6 (100)	4 (66.7)	25 (100)	17 (68.0)
Leukopenia	4 (66.7)	2 (33.3)	6 (100)	3 (50.0)	4 (57.1)	2 (28.6)	4 (66.7)	2 (33.3)	18 (72.0)	9 (36.0)
Neutropenia	5 (83.3)	4 (66.7)	5 (83.3)	3 (50.0)	2 (28.6)	2 (28.6)	5 (83.3)	4 (66.7)	17 (68.0)	13 (52.0)
Alopecia	1 (16.7)	0	2 (33.3)	0	0	0	6 (100)	0	9 (36.0)	0
Anemia	3 (50.0)	0	1 (16.7)	1 (16.7)	2 (28.6)	1 (14.3)	3 (50.0)	0	9 (36.0)	2 (8.0)
Stomatitis	2 (33.3)	0	2 (33.3)	0	2 (28.6)	0	3 (50.0)	0	9 (36.0)	0
Lymphopenia	1 (16.7)	0	3 (50.0)	2 (33.3)	1 (14.3)	0	3 (50.0)	2 (33.3)	8 (32.0)	4 (16.0)
Thrombocytopenia	1 (16.7)	0	2 (33.3)	0	1 (14.3)	0	4 (66.7)	0	8 (32.0)	0
ALT increased	2 (33.3)	0	0	0	1 (14.3)	0	4 (66.7)	0	7 (28.0)	0
AST increased	2 (33.3)	0	2 (33.3)	0	1 (14.3)	0	2 (33.3)	0	7 (28.0)	0
Infusion-related reaction	0	0	2 (33.3)	0	2 (28.6)	0	2 (33.3)	0	6 (24.0)	0
Pyrexia	2 (33.3)	0	2 (33.3)	0	1 (14.3)	0	1 (16.7)	0	6 (24.0)	0
Rash	2 (33.3)	0	1 (16.7)	0	2 (28.6)	0	1 (16.7)	0	6 (24.0)	0
Nausea	3 (50.0)	0	1 (16.7)	0	0	0	1 (16.7)	0	5 (20.0)	0
Decreased appetite	0	0	1 (16.7)	0	1 (14.3)	0	2 (33.3)	0	4 (16.0)	0
Peripheral sensory neuropathy	2 (33.3)	0	1 (16.7)	0	0	0	1 (16.7)	0	4 (16.0)	0
Amylase increased	1 (16.7)	1 (16.7)	1 (16.7)	0	0	0	0	0	2 (8.0)	1 (4.0)
Diarrhea	0	0	0	0	1 (14.3)	1 (14.3)	1 (16.7)	0	2 (8.0)	1 (4.0)
Febrile neutropenia	1 (16.7)	1 (16.7)	0	0	0	0	1 (16.7)	1 (16.7)	2 (8.0)	2 (8.0)
Lipase increased	1 (16.7)	1 (16.7)	1 (16.7)	1 (16.7)	0	0	0	0	2 (8.0)	2 (8.0)

Abbreviations: ALT, alanine aminotransferase; AST, aspartate aminotransferase; E7389-LF, eribulin liposomal formulation; TEAE, treatment-emergent adverse event.

^a^E7389-LF dose plus nivolumab 360 mg.

^b^E7389-LF dose plus nivolumab 240 mg.

^c^No grade 5 severity treatment-related TEAEs were observed.

Three patients (12.0%) had at least one TEAE leading to study drug discontinuation, 2 of whom were in the E7389-LF every-3-weeks cohorts (one case of hypoxia in the 2.1 mg/m^2^ cohort and one case of enterocolitis in the 1.7 mg/m^2^ cohort) and 1 patient in the E7389-LF 1.4 mg/m^2^ every-2-weeks cohort (pneumonia and pneumothorax). Three patients in the E7389-LF every-3-weeks cohorts (25.0%) and 4 in the E7389-LF every-2-weeks cohorts (30.8%) had at least one TEAE resulting in study drug interruption. Overall, 3 patients (12.0%) had at least one TEAE leading to reduction of their E7389-LF dose: 2 patients had neutropenia leading to dose reduction in the E7389-LF 2.1 mg/m^2^ every-3-weeks (*n* = 1) and E7389-LF 1.1 mg/m^2^ every-2-weeks (*n* = 1) cohorts and 1 patient had peripheral sensory neuropathy leading to dose reduction in the E7389-LF 1.7 mg/m^2^ every-3-weeks cohort. The every-3-weeks schedule also had a higher relative dose intensity of E7389-LF than the every-2-weeks schedule (mean dose intensities, 91.8% vs. 78.3%, respectively).

### Pharmacokinetic Analyses

The plasma eribulin concentration showed a monophasic decline after the administration of E7389-LF on C1D1 (Supplementary Fig. S2). The mean C_max_ and AUC values increased dose-dependently; the mean t_1/2_ values were 19.9–22.5 hours and were not dose dependent (Supplementary Table S4). In 2 patients in the E7389-LF 1.4 mg/m^2^ every-2-weeks cohort, unique plasma eribulin concentration profiles (two-phasic decline of plasma eribulin concentration and lower AUC values) were observed. The cause of this difference is unknown but their pharmacokinetic profiles were considered to be outliers because their profiles were remarkably different from those of the other patients in this study and the previous E7389-LF monotherapy study (10). Therefore, the 2 patients were excluded from the summary of plasma concentration profiles and pharmacokinetic parameters of eribulin. The mean serum nivolumab concentrations just before the completion of infusion (C_max_) on C1D1 were 65.0–66.6 μg/mL and 105–114 μg/mL at the 240 mg and 360 mg dose, respectively (Supplementary Table S5).

### Efficacy

**FIGURE 1:**
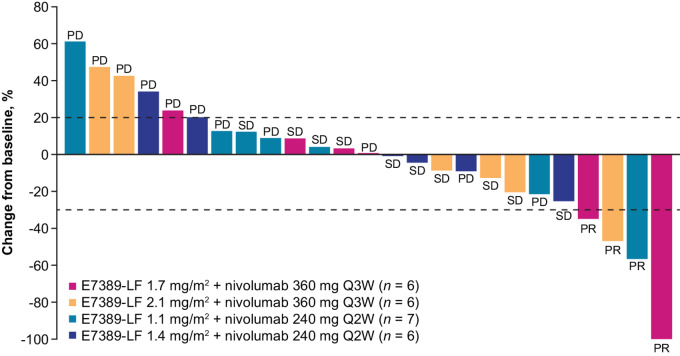
Maximum percentage change from baseline in sums of tumor diameters and best overall response per RECIST v1.1. E7389-LF, eribulin liposomal formulation; PD, progressive disease; PR, partial response; Q#W, every # weeks; RECIST v1.1, Response Evaluation Criteria In Solid Tumors, version 1.1; SD, stable disease.

**FIGURE 2:**
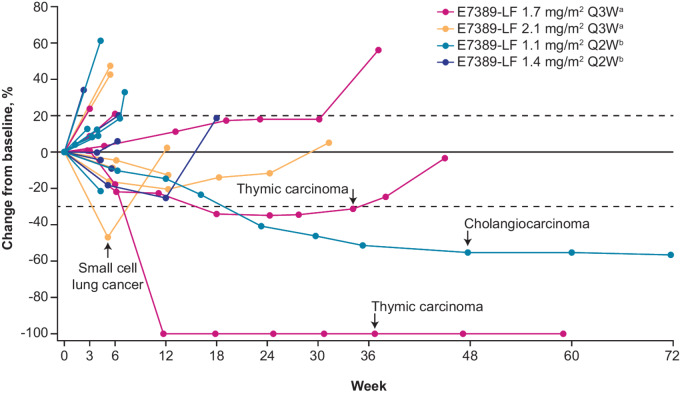
Percentage changes from baseline in sums of target lesion diameters over time per RECIST v1.1 by investigator assessment. Tumor types of patients with at least a 30% reduction at any timepoint are listed. ^a^Plus nivolumab 360 mg every 3 weeks; ^b^plus nivolumab 240 mg every 2 weeks. Q#W, every # weeks; RECIST v1.1, Response Evaluation Criteria In Solid Tumors version 1.1.

Overall, a PR was observed in 4 of the 25 patients (16.0%; Table 3; Fig. 1): 3 patients were in the E7389-LF every-3-weeks dosing cohorts (2 patients with thymic carcinoma who received E7389-LF 1.7 mg/m^2^ and 1 patient with small cell lung cancer who received E7389-LF 2.1 mg/m^2^) and 1 patient with cholangiocarcinoma in the E7389-LF 1.1 mg/m^2^ every-2-weeks cohort. All 4 of these patients had received prior anticancer therapy. One of the patients with thymic carcinoma had received carboplatin plus paclitaxel, S-1, gemcitabine, an investigational drug in combination with an ICI, and an additional investigational drug; the other patient with thymic carcinoma had received carboplatin plus paclitaxel. The patient with small cell lung cancer had received cisplatin plus etoposide, atezolizumab plus carboplatin plus etoposide, amrubicin, and cisplatin plus irinotecan. The one patient with cholangiocarcinoma had received cisplatin plus gemcitabine, resminostat plus S-1, and an investigational drug. The overall DCR was 48.0% (95% CI: 27.8–68.7; Table 3). Changes in patients’ tumor sizes (sums of target lesion diameters) from baseline over time are shown in Fig. 2.

**TABLE 3:** Summary of tumor responses (per Investigator Review by RECIST v1.1)

	Every 3 weeks^a^		Every 2 weeks^b^		
Tumor response	E7389-LF 1.7 mg/m^2^ *n* = 6	E7389-LF 2.1 mg/m^2^ *n* = 6	E7389-LF 1.1 mg/m^2^ *n* = 7	E7389-LF 1.4 mg/m^2^ *n* = 6	Total (*N* = 25)
**BOR, *n* (%)**					
CR	0	0	0	0	0
PR	2 (33.3)	1 (16.7)	1 (14.3)	0	4 (16.0)
SD^c^	1 (16.7)	3 (50.0)	1 (14.3)	3 (50.0)	8 (32.0)
PD	3 (50.0)	2 (33.3)	4 (57.1)	3 (50.0)	12 (48.0)
Unknown/NE	0	0	1 (14.3)	0	1 (4.0)
**ORR, *n* (%)** **^d^**	2 (33.3)	1 (16.7)	1 (14.3)	0	4 (16.0)
95% CI	4.3–77.7	0.4–64.1	0.4–57.9	0.0–45.9	4.5–36.1
**DCR, *n* (%)** **^e^**	3 (50.0)	4 (66.7)	2 (28.6)	3 (50.0)	12 (48.0)
95% CI	11.8–88.2	22.3–95.7	3.7–71.0	11.8–88.2	27.8–68.7

Abbreviations: BOR, best overall response; CI, confidence interval; CR, complete response; DCR, disease control rate; E7389-LF, eribulin liposomal formulation; NE, not evaluable; ORR, objective response rate; PD progressive disease; PR, partial response; RECIST v1.1, Response Evaluation Criteria in Solid Tumors version 1.1; SD, stable disease.

^a^E7389-LF dose plus nivolumab 360 mg.

^b^E7389-LF dose plus nivolumab 240 mg.

^c^≥5 weeks after starting treatment on C1D1.

^d^CR + PR.

^e^CR + PR + SD.

### Biomarker Analyses

**FIGURE 3:**
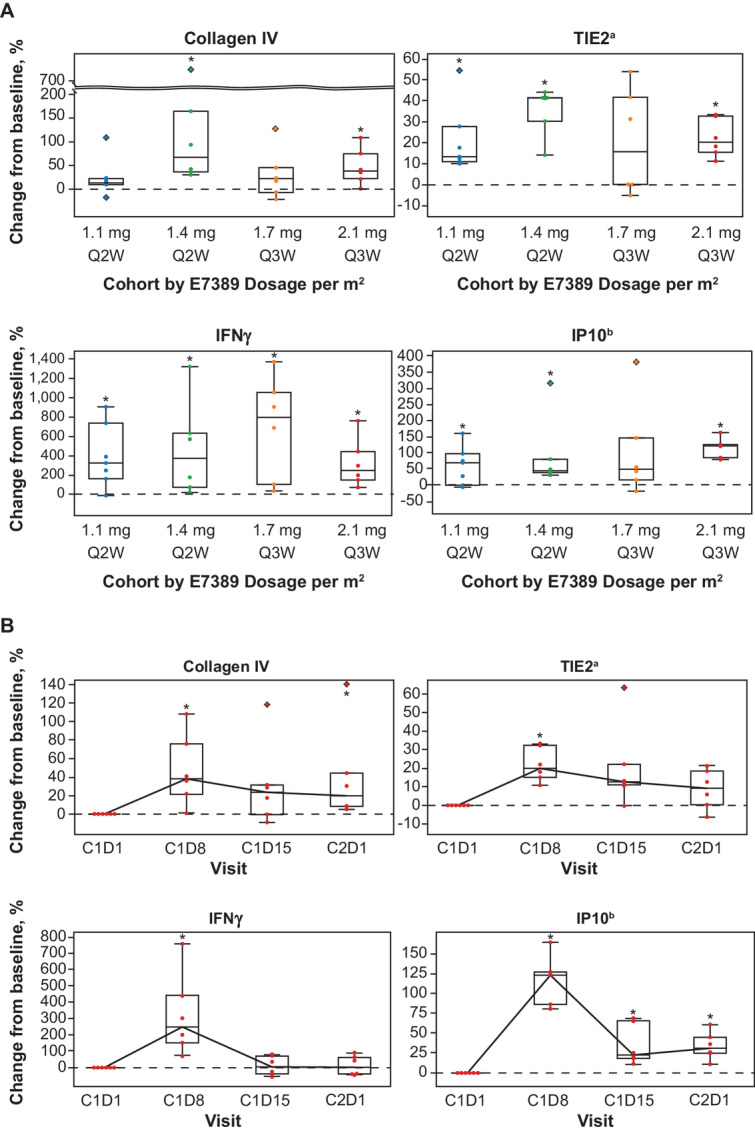
Analyses of changes in key biomarkers from baseline to C1D8 in all dosing cohorts (**A**) and from C1D1 to C2D1 in the E7389-LF 2.1 mg/m^2^ every-3-weeks cohort (**B**). Boxes indicate the median and quartiles, the whiskers represent the largest or smallest values within 1.5 times the interquartile range, and * denotes an unadjusted *P* value of < 0.05 (Wilcoxon signed-rank test); the diamond shape ◊ denotes outlier. Biomarkers also known as: ^a^TEK; ^b^CXCL10. CXCL, C-X-C motif chemokine ligand; C#, cycle; D#, day #; IP10, IFNγ-induced protein 10; Q#W, every # week; TEK, TEK receptor tyrosine kinase; TIE2, tyrosine kinase immunoglobulin and EGF homology domains 2.

In all four cohorts, increases in pharmacodynamic markers of focus from baseline to C1D8 suggested vascular remodeling activity and enhancement of antitumor immunity via IFNγ signaling (Fig. 3A). Similar trends of changes in vasculature-related markers and immune-related markers were observed over time in all four cohorts. In the E7389-LF 2.1 mg/m^2^ every-3-weeks cohort, immune-related marker IFNγ increased in concentration from C1D1 to C1D8 (Fig. 3B). Vasculature-related markers collagen IV and TIE2, as well as IP10, increased from C1D1 to C2D1. Changes in biomarker levels from baseline to C1D8 and C1D15 in all dosing cohorts are available in Supplementary Table S6.

Among patients who had available tumor samples at screening and at C2D1, 10 patients had an immune-desert or immune-excluded phenotype at screening (Supplementary Table S7). Of these patients, 4 had their phenotype changed at C2D1 from immune-desert or immune-excluded to immune-inflamed. These included 1 patient in the E7389-LF 1.1 mg/m^2^ every-2-weeks cohort with cholangiocarcinoma (immune-desert), 2 patients in the E7389-LF 1.4 mg/m^2^ every-2-weeks cohort with ovarian cancer (*n* = 1; immune-desert) and urothelial carcinoma (*n* = 1; immune-excluded), and 1 patient in the E7389-LF 1.7 mg/m^2^ every-3-weeks cohort with ovarian cancer (immune-desert; Supplementary Table S7). All 4 of these patients had a measured CPS of 0 at baseline (Supplementary Table S7). The 1 patient with cholangiocarcinoma (in the E7389-LF 1.1 mg/m^2^ every-2-weeks cohort) who had their immune phenotype changed had a PR (Supplementary Fig. S3), while the other patients with a changed phenotype did not have a response (Supplementary Table S7). In comparison, another patient with cholangiocarcinoma had their phenotype changed from immune-desert to immune-excluded and had SD as their best response (Supplementary Table S7; Supplementary Fig. S3). The results of the gene analysis showed that 3 of 4 patients with a PR tended to show a higher expression of immune cell–type genes at baseline, or a higher fold change of immune cell–type genes from baseline to C2D1 (Supplementary Fig. S4).

As a result of the DLT, safety (including relative dose intensity), pharmacokinetic, efficacy, and biomarker analyses, the RP2D was determined to be E7389-LF 2.1 mg/m^2^ every 3 weeks plus nivolumab 360 mg every 3 weeks.

## Discussion

E7389-LF plus nivolumab was tolerable in patients with advanced solid tumors, with antitumor effects seen in both the every-3-weeks and every-2-weeks schedules. DLTs occurred in 3 patients, and all were resolved. All patients had at least one treatment-related TEAE, most commonly leukopenia and neutropenia, that were related to E7389-LF specifically. There were no substantial changes in the overall pharmacokinetic profiles of E7389-LF and nivolumab compared with previous clinical studies of E7389-LF (10) and nivolumab (21, 22), respectively. Specifically, E7389-LF 2.1 mg/m^2^ every 3 weeks plus nivolumab 360 mg every 3 weeks was considered to be tolerable as no DLTs occurred in this dose cohort. Considering this, along with the similar safety and efficacy between cohorts, dose-dependent pharmacokinetic and biomarker profiles, and higher relative dose intensity in the every-3-weeks cohorts versus the every-2-weeks cohorts (including relative dose intensity), E7389-LF 2.1 mg/m^2^ every 3 weeks plus nivolumab 360 mg every 3 weeks was determined to be the RP2D.

Incidence of leukopenia and neutropenia, as well as the occurrence of the neutrophil count nadir (caused by bone marrow suppression) around days 8 to 15 for the combination of E7389-LF plus nivolumab across dose schedules was consistent with studies of E7389-LF monotherapy (10–12). In a phase I study of E7389-LF monotherapy (the dose-escalation part of Study 114), neutropenia was the most common grade ≥3 severity TEAE (66.7%) in Japanese patients with solid tumors, and the neutrophil nadir occurred during days 8–15 (10). Similar patterns were also seen in Japanese patients with breast cancer and with gastric cancer, treated with E7389-LF monotherapy at the MTD [E7389-LF 2.3 mg/m^2^ (eribulin mesylate equivalent) every 3 weeks] in Study 114 (11, 12). Of interest, in those analyses in E7389-LF monotherapy, patients who received prophylactic pegfilgrastim had lower incidences of neutropenia and febrile neutropenia than patients who did not receive prophylactic treatment.

While care should be taken in drawing comparisons between clinical trials, the toxicity profile of the E7389-LF plus nivolumab combination appears comparable with that of E7389-LF alone (10–12). The higher relative dose intensity seen in the every-3-weeks cohorts may have been a result of skipped doses at day 15 in the every-2-weeks cohorts because of low neutrophil counts. A greater proportion of TEAEs resulting in study drug interruption were observed in the every-2-weeks schedule (30.8%) compared with the every-3-weeks schedule (25.0%). Use of the every-3-weeks dosing schedule may help to avoid skipped doses due to this temporary drop in neutrophil count.

Clear changes in expression of collagen IV, TIE2, IFNγ, and IP10 from baseline suggests that E7389-LF plus nivolumab treatment results in vascular remodeling activity and enhancement of antitumor immunity via IFNγ signaling. Vasculature marker changes may be due to E7389-LF while changes in IFN-related biomarkers may be a result of ICI-combination therapy. The observed changes in immune phenotype from desert or excluded to inflamed suggest that E7389-LF might enhance immune activity in immune-insufficient types of tumors via a vascular remodeling effect, leading to passage of tumor-infiltrating lymphocytes into tumors. Of 2 patients with cholangiocarcinoma assessed, the patient who had their phenotype changed to “immune-inflamed” had a partial response; this was despite a very low tumor infiltration (PD-L1 CPS of 0) at baseline. Similarly, a preclinical study of E7389-LF and an anti-PD-1 therapy has suggested that E7389-LF shows unique immunomodulatory properties, including increasing the population of T cells and natural killer cells (19). These findings are limited by the small number of patients in this part; however, a larger population (including patients with gastric cancer, esophageal cancer, and small cell lung cancer) in the ongoing phase II part of this study will help to validate these outcomes. In addition, further investigation is needed to clearly illustrate enhancement of IFN signaling by the combination.

As the results from this phase Ib analysis demonstrated promising antitumor activity with no new or unexpected safety signals, the phase II data are expected to shed further light on the potential success of E7389-LF plus nivolumab in the treatment of patients with solid tumors.

## Supplementary Material

Supplementary Methods 1 Supplementary Methods

Supplementary Table 1 Supplementary Table 1. Dose-Limiting Toxicities

Supplementary Table 2 Supplementary Table 2. Representativeness of Study Patients

Supplementary Table 3 Supplementary Table 3. Safety Summary by E7389-LF Dose

Supplementary Table 4 Supplementary Table 4. Pharmacokinetic Parameters After Administration of E7389-LF

Supplementary Table 5 Supplementary Table 5. Summary of Serum Nivolumab Concentrations After the First Dose

Supplementary Table 6 Supplementary Table 6. Analyses of Median Changes in Plasma Protein Expression of Biomarker Analytes From Baseline to C1D8 and C1D15 in All Dosing Cohorts

Supplementary Table 7 Supplementary Table 7. Tumor Biomarkers: Assessment of Immune Phenotypes

Supplementary Figure 1 S1. Box-and-Whisker Plot of Neutrophil Measurements During Cycle 1 for the (A) Q3W and (B) Q2W Dosing Groups.

Supplementary Figure 2 S2. Linear (A) and Semi-logarithmic (B) Plasma Concentration Profiles of Eribulin After Administration of E7389-LF.

Supplementary Figure 3 S3. Imaging of 2 Patients with Cholangiocarcinoma During Treatment.

Supplementary Figure 4 S4. Gene Expression Profiling in Biopsy Tissues
