# Supplementary material for: Phase Ib/II Study of a Liposomal Formulation of Eribulin (E7389-LF) plus Nivolumab in Patients with Advanced Solid Tumors: Results from Phase Ib
Source: Cancer Res Commun. 2023 Jul 10;3(7):1189–99. doi: 10.1158/2767-9764.CRC-22-0401 (PMC10332326; doi:10.1158/2767-9764.CRC-22-0401)
Supplement: Supplementary Methods 1 — Supplementary Methods [file crc-22-0401-s01.pdf]

Supplementary Material

Supplementary Methods

Inclusion/Exclusion Criteria

Inclusion Criteria

1. Phase 1b part only: patients with advanced, nonresectable, or recurrent solid tumor for which no alternative standard therapy or no effective therapy exists (patients who will be the candidate of treatment by nivolumab monotherapy as standard therapy is acceptable).

2. Phase 2 part only: nonresectable gastric cancer (GC), esophageal cancer (EGC), or small cell lung cancer (SCLC) patients with confirmed diagnosis who showed disease progression by investigator's assessment during or after 1st-line chemotherapy (2nd-line chemotherapy for GC) and did not receive any other systemic chemotherapy for advanced/recurrent disease.

3. Phase 2 part only: patients who received below as prior chemotherapy:
   a. GC: combination therapy including platinum agent and fluoropyrimidine as 1st-line therapy, followed by 2nd line therapy including taxane
   b. EGC: combination therapy including platinum agent and fluoropyrimidine (without taxane) as 1st-line therapy
   c. SCLC: combination therapy including platinum agent as 1st-line therapy.

4. Patients who have accessible tumors for biopsy and agree with tumor biopsy pretreatment and posttreatment of study drug (if a pretreatment biopsy cannot be obtained due to safety issue, then an archival tumor tissue sample may be submitted).

5. Life expectancy of ≥12 weeks.

6. Eastern Cooperative Oncology Group performance status 0–1.

7. Age ≥20 years at the time of informed consent.

8. All adverse events (AEs) due to previous anti-cancer therapy have either returned to Grade 0–1 except for alopecia and Grade 2 peripheral neuropathy (renal/bone marrow/liver/pancreatic function should meet the inclusion criteria).

9. Adequate washout period from the end of prior therapy to cycle 1 day 1 (cycle # day #; C#D#):
   a. Noncytotoxic anticancer therapy: 4 weeks (or 5 × half-life, whichever shorter) or more
   b. Cytotoxic anticancer therapy and radiotherapy: 3 weeks or more (radiotherapy for brain metastasis in SCLC patients: 2 weeks or more)
   c. Anticancer therapy with antibody: 4 weeks or more
   d. Any investigational drug or device: 4 weeks or more
   e. Blood/platelet transfusion or granulocyte colony-stimulating factor (GCSF): 2 weeks or more
   f. Live/attenuated vaccine: 4 weeks or more.

10. Adequate renal function defined as serum creatinine ≤1.5 × upper limit of normal (ULN) (>1.5 × ULN with creatinine clearance ≥40 mL/min per the Cockcroft and Gault formula is acceptable).

11. Adequate bone marrow function:
    a. Absolute neutrophil count (ANC) ≥2000/mm^3
    b. Platelets ≥100 000/mm^3
    c. Hemoglobin ≥8.5 g/dL.

12. Adequate liver function:
    a. Adequate blood coagulation function as evidenced by an International Normalized Ratio (INR) ≤1.5
    b. Total bilirubin ≤1.5 ×ULN (≤ 3.0 × ULN for patients with Gilbert's syndrome)
    c. Alkaline phosphatase, alanine aminotransferase (ALT), and aspartate aminotransferase (AST) ≤3 × ULN (≤5 × ULN for patients with liver tumor lesion).

13. Adequate pancreatic function defined as amylase and lipase ≤1.5 ×ULN (≤ 3.0 × ULN for patients with pancreatic tumor lesion).

14. Phase 2 part only: At least 1 measurable lesion based on RECIST v1.1 (Lesions that have had radiotherapy or loco-regional therapies must show evidence of progressive disease to be deemed a measurable lesion).

15. Willing and able to give informed consent and comply with all aspects of the protocol.

Exclusion Criteria

1. Any of cardiac conditions as follows:
    a. Heart failure of New York Heart Association Class II or above
    b. Unstable ischemic heart disease (myocardial infarction within 6 months prior to C1D1, or angina requiring use of nitrates more than once weekly)
    c. Prolongation of corrected QT (QTcF) interval to >480 ms.

2. History of hypersensitivity reaction by liposomal formulation agent.

3. Major surgery within 21 days prior to C1D1.

4. Previous treatment with eribulin (including E7389-LF).

5. Previous treatment with any anti-PD-1, anti-PD-L1, anti-PD-L2, anti-CD137, or anti-CTLA-4 antibody, or any other antibody or drug specifically targeting T cell co-stimulation or checkpoint, or cancer vaccine therapy which showed Grade ≥3 immune-related AE (irAE) or needed treatment discontinuation by any Grade of irAE.

6. Known intolerance to the study drug or any of the excipients, or any antibody agent.

7. Known to be HIV positive or known to have AIDS.

8. Active viral hepatitis (HBV or HCV) as demonstrated by positive HBV surface (HBs) antigen or negative HBs antigen with positive HBs or HBc antibody and positive HBV-DNA, or positive HCV-RNA.

9. Active infection requiring systemic therapy within 14 days of C1D1.

10. Diagnosed with meningeal carcinomatosis.
11. Patients with brain or subdural metastases or invasion are not eligible, unless they have completed local therapy and have discontinued the use of corticosteroids for this indication for at least 4 weeks (2 weeks for SCLC patients) before C1D1. Any signs (eg, radiologic) or symptoms of brain metastases must be stable for at least 4 weeks (2 weeks for SCLC patients) before C1D1.
12. Pulmonary lymphangitic involvement that results in pulmonary dysfunction requiring active treatment, including the use of oxygen.
13. Patients with any active, known, or suspected autoimmune disease, with the following exceptions:
    a. Patients with vitiligo, type 1 diabetes mellitus, resolved childhood asthma or atopy
    b. Patients with suspected autoimmune thyroid disorders may be enrolled if they are currently euthyroid or with residual hypothyroidism requiring only hormone replacement.
14. Patients with active or history of interstitial pneumonia or pulmonary fibrosis as diagnosed clinically or by imaging. Patients with radiation pneumonitis will be eligible if stabilization from fibrosis is confirmed and there is no concern for recurrence.
15. Any history of organ transplant that requires use of immune suppressive agents.
16. Patients with a condition requiring systemic treatment with either corticosteroids (>10 mg/day prednisone equivalent) or other immunosuppressive medications within 14 days of C1D1. Inhaled or topical corticosteroids (with minimal systemic absorption) are permitted in the absence of active autoimmune disease.
17. Phase 2 part only: history of active malignancy (except for primary tumor, or definitively treated melanoma in situ, basal or squamous cell carcinoma of the skin, carcinoma in situ of the bladder or cervix, or early stage gastric/colorectal/head and neck cancer) within the past 24 months prior to C1D1.
18. Evidence of clinically significant disease/status (eg, cardiac, respiratory, gastrointestinal, renal disease) that in the opinion of the investigator(s) could affect the patient's safety or interfere with the study assessments.
19. Women who are breastfeeding or pregnant at Screening or Baseline (as documented by a positive beta-human chorionic gonadotropin [ß-hCG] or hCG test). A separate baseline assessment is required if a negative screening pregnancy test was obtained >72 hours before C1D1.
20. Men with impregnation potential or women of childbearing potential who do not (or whose partner does not) agree with medically effective method of contraception throughout the entire study period and for 5 months (3 months for males) after study drug discontinuation.
