# Supplementary material for: Phase Ib/II Study of a Liposomal Formulation of Eribulin (E7389-LF) plus Nivolumab in Patients with Advanced Solid Tumors: Results from Phase Ib
Source: Cancer Res Commun. 2023 Jul 10;3(7):1189–99. doi: 10.1158/2767-9764.CRC-22-0401 (PMC10332326; doi:10.1158/2767-9764.CRC-22-0401)
Supplement: Supplementary Table 1 — Dose-Limiting Toxicities [file crc-22-0401-s02.pdf]

Supplementary Table S1. Dose-Limiting Toxicities

Hematological toxicities	- Grade 4 neutropenia lasting ≥8 days<br>- Grade 3–4 febrile neutropenia<br>- Grade 4 thrombocytopenia<br>- Grade 3 thrombocytopenia lasting ≥8 days, with bleeding or requiring platelet transfusion<br>- Grade 3–4 lymphopenia with clinical manifestations<br>- Grade 4 anemia<br>- Grade 3 anemia requiring transfusion<br>- Grade 5 event
Nonhematological toxicities	- Grade 3–4 hyper- or hypothyroidism not controlled by optimal treatment<br>- Grade 3 glucose intolerance not controlled by glucose controlling agents<br>- Grade 4–5 event<br>- Clinically significant grade 3 event (except for fatigue, diarrhea, nausea and vomiting resolved to grade 0–2 by optimal supportive care within 3 days)<br>- Grade 4 amylase or lipase elevations<br>- Clinically significant grade 3–4 abnormal clinical laboratory values lasting ≥8 days<br>- Grade 2 uveitis, eye pain, or blurred vision that does not respond to topical therapy and does not improve to grade 0–1 within 6 weeks or requires systemic treatment
Q2W dosing schedule only	Dose-skip E7389-LF on day 15 if meeting any of the below criteria:<br>- Absolute neutrophil count <1000/mm^3<br>- Platelet count <75 000/mm^3<br>- Nonhematologic toxicity of E7389-LF not recovered to grade 0–2 (except for abnormal clinical laboratory values not clinically significant)
Notes:<br>If the dose of nivolumab on C1D15 (Q2W) was skipped by meeting dose interruption criteria, investigators and the sponsor discussed and decided whether the events should be classified as DLTs and whether the patient was evaluable for DLT, with consultation with external safety advisor if needed. If the dose of study drug on C1D15 was skipped due to an AE not related to study drug, this case was not regarded as a DLT, and an additional patient was enrolled.	

AE, adverse event; C# D#, cycle # day #; DLT, dose-limiting toxicity; E7389-LF, eribulin liposomal formulation; Q2W, every 2 weeks.
