# Supplementary material for: Phase Ib/II Study of a Liposomal Formulation of Eribulin (E7389-LF) plus Nivolumab in Patients with Advanced Solid Tumors: Results from Phase Ib
Source: Cancer Res Commun. 2023 Jul 10;3(7):1189–99. doi: 10.1158/2767-9764.CRC-22-0401 (PMC10332326; doi:10.1158/2767-9764.CRC-22-0401)
Supplement: Supplementary Table 2 — Representativeness of Study Patients [file crc-22-0401-s03.pdf]

Supplementary Table S2. Representativeness of Study Patients

**Cancer Types**	Solid tumors (including ovarian cancer, thymic carcinoma, gastric cancer, cholangiocarcinoma, colorectal cancer, neuroendocrine carcinoma, small cell lung cancer, among others).
**Considerations related to:**	
Sex/gender	Most of the patients in our trial (64.0%) were male. This trend is similar to findings of the 2020 GLOBOCAN report, where males in Japan had an age-standardized incidence rate of cancer of 328.1 compared with 253.8 for females.[a]
Age	The median age of onset in our study was 55.0 years. Compared to previous studies, this median age was similar to, or younger than, the average age of Japanese patients (at diagnosis or during the study) with ovarian cancer,[b] thymic carcinoma,[c] and gastric cancer,[d,e] which were among the most frequently represented cancer types in our trial.
Race/ethnicity/geography	All patients in our trial were Japanese. Several of the most frequently appearing cancer types in our trial (including gastric cancer, colorectal cancer, and ovarian cancer) correspond to the most common cancer sites reported among Japanese patients.[a]
**Overall representativeness of this study**	Generally, characteristics of patients in our study share several factors with Japanese adults diagnosed with cancer. However, given the broad range of cancer types enrolled, as well as the small population size, our findings should be validated in a larger sample size enrolling patients from different countries with specific cancer types.

[a]Japan – Global Cancer Observatory. GLOBOCAN 2020. Accessed January 13, 2023. https://gco.iarc.fr/today/data/factsheets/populations/392-japan-fact-sheets.pdf; [b]Enomoto T, Aoki D, Hattori K, Jinushi M, Kigawa J, Takeshima N, et al. The first Japanese nationwide multicenter study of BRCA mutation testing in ovarian cancer: CHARacterizing the cross-sectionaL approach to Ovarian cancer geneTic TEsting of BRCA (CHARLOTTE). *Int J Gynecol Cancer*. **2019**;29:1043-1049; [c]Koizumi T, Otsuki K, Tanaka Y, Noguchi T, Fukushuima T, Kobayashi T, et al. National incidence and initial therapy for thymic carcinoma in Japan: based on analysis of hospital-based cancer registry data, 2009-2015. *Jpn J Clin Oncol* **2020**;50(4):434-439; [d]Katai H, Ishikawa T, Akazawa K, Isobe Y, Miyashiro I, Oda I, et al. Five-year survival analysis of surgically resected gastric cancer cases in Japan: a retrospective analysis of more than 100,000 patients from the nationwide registry of the Japanese Gastric Cancer Association (2001–2007). *Gastric Cancer* **2018**;21:114-154; [e]Yoshida T, Ogura G, Tanabe M, Hayashi T, Ohbayashi C, Azuma M, et al. Clinicopathological features of PD-L1 protein expression, EBV positivity, and MSI status in patients with advanced gastric and esophagogastric junction adenocarcinoma in Japan. *Cancer Biol Ther* **2022**;23(1):191-200.
