# Supplementary material for: Phase Ib/II Study of a Liposomal Formulation of Eribulin (E7389-LF) plus Nivolumab in Patients with Advanced Solid Tumors: Results from Phase Ib
Source: Cancer Res Commun. 2023 Jul 10;3(7):1189–99. doi: 10.1158/2767-9764.CRC-22-0401 (PMC10332326; doi:10.1158/2767-9764.CRC-22-0401)
Supplement: Supplementary Table 3 — Safety Summary by E7389-LF Dose [file crc-22-0401-s04.pdf]

Supplementary Table S3. Safety Summary by E7389-LF Dose

Parameters, n (%)	Every 3 weeks[a]		Every 2 weeks[b]		Total
	**E7389-LF 1.7 mg/m^2 *n* = 6**	**E7389-LF 2.1 mg/m^2 *n* = 6**	**E7389-LF 1.1 mg/m^2 *n* = 7**	**E7389-LF 1.4 mg/m^2 *n* = 6**	***N* = 25**
**Any TEAEs**	6 (100)	6 (100)	7 (100)	6 (100)	25 (100)
Grade 3–5	5 (83.3)	4 (66.7)	5 (71.4)	5 (83.3)	19 (76.0)
**Treatment-related TEAEs**	6 (100)	6 (100)	7 (100)	6 (100)	25 (100)
Grade 3–4	5 (83.3)	4 (66.7)	4 (57.1)	4 (66.7)	17 (68.0)
**SAEs**	2 (33.3)	1 (16.7)	2 (28.6)	2 (33.3)	7 (28.0)
Pneumonia	0	0	1 (14.3)	1 (16.7)	2 (8.0)
Cholangitis	1 (16.7)	0	0	0	1 (4.0)
Enterocolitis	1 (16.7)	0	0	0	1 (4.0)
Hypoxia	0	1 (16.7)	0	0	1 (4.0)
Malignant pleural effusion	0	0	0	1 (16.7)	1 (4.0)
Metastases to meninges	0	0	1 (14.3)	0	1 (4.0)
Pneumothorax	0	0	0	1 (16.7)	1 (4.0)
**Treatment-related SAEs**	1 (16.7)	0	0	0	1 (4.0)
Enterocolitis	1 (16.7)	0	0	0	1 (4.0)
**DLTs**					DLT evaluable (*n* = 24)[c]
Febrile neutropenia	1 (16.7)	0	0	1 (16.7)	2 (8.3)[d]
Neutropenia	0	0	1 (16.7)[c]	0	1 (4.2)[d]

[a]E7389-LF dose plus nivolumab 360 mg; [b]E7389-LF dose plus nivolumab 240 mg; [c]1 patient in the E7389-LF 1.1 m^2 Q2W cohort was not evaluable for DLTs; [d]percentage is out of the 24 patients who were evaluable for DLTs.

DLT, dose-limiting toxicity; E7389-LF, eribulin liposomal formulation; Q2W, every 2 weeks; SAE, serious adverse event; TEAE, treatment-emergent adverse event.
