# Supplementary material for: Phase Ib/II Study of a Liposomal Formulation of Eribulin (E7389-LF) plus Nivolumab in Patients with Advanced Solid Tumors: Results from Phase Ib
Source: Cancer Res Commun. 2023 Jul 10;3(7):1189–99. doi: 10.1158/2767-9764.CRC-22-0401 (PMC10332326; doi:10.1158/2767-9764.CRC-22-0401)
Supplement: Supplementary Table 4 — Pharmacokinetic Parameters After Administration of E7389-LF [file crc-22-0401-s05.pdf]

Supplementary Table S4. Pharmacokinetic Parameters After Administration of E7389-LF

E7389-LF Dose level[a]	C_{max} (ng/mL)	$AUC_{(0-t)}$ (ng•h/mL)	$AUC_{(0-inf)}$ (ng•h/mL)	t_{max} (h)	$t_{1/2}$ (h)	CL ($L/h/m^2$)	V_{ss} (L/m^2)
1.1 mg/m^2 (*n* = 7)	518 ± 73.8	17300 ± 3690	17400 ± 3760	1.48 (1.00–4.92)	21.9 ± 3.31	0.0598 ± 0.0133	1.86 ± 0.299
1.4 mg/m^2 (*n* = 4)[b]	675 ± 75.1	21000 ± 5740	21200 ± 5840	0.96 (0.92–1.05)	22.5 ± 1.28	0.0596 ± 0.0140	1.84 ± 0.339
1.7 mg/m^2 (*n* = 6)	869 ± 68.4	26900 ± 5070	27100 ± 5150	1.24 (0.95–4.92)	21.3 ± 1.95	0.0570 ± 0.0102	1.68 ± 0.179
2.1 mg/m^2 (*n* = 6)	979 ± 145	27200 ± 3910	27300 ± 3930	1.95 (0.98–3.60)	19.9 ± 1.85	0.0671 ± 0.00977	1.92 ± 0.284

Data are shown as mean ± standard deviation except t_{max}. For t_{max}, median (minimum–maximum) is shown.

[a]Dose levels are shown as eribulin mesylate. Dose levels as free eribulin are 1.0, 1.2, 1.5, 1.8 mg/m^2, respectively; [b]2 patients in the 1.4 mg/m^2 cohort showed unique plasma eribulin concentration profiles, and thus were excluded from this row. The parameters of the excluded 2 patients were as follows; C_{max}: 444 and 364 ng/mL; $AUC_{(0-inf)}$: 2110 and 1390 ng•h/mL, respectively.

AUC, area under the plasma concentration–time curve; $AUC_{(0-t)}$, AUC from zero time to time of last quantifiable concentration; $AUC_{(0-inf)}$, AUC from zero time extrapolated to infinite time; CL, total clearance; C_{max}, maximum plasma concentration; E7389-LF, eribulin liposomal formulation; $t_{1/2}$, terminal elimination phase half-life; t_{max}, time at which the maximum plasma concentration occurs; V_{ss}, volume of distribution at steady state.
