# Supplementary material for: Phase Ib/II Study of a Liposomal Formulation of Eribulin (E7389-LF) plus Nivolumab in Patients with Advanced Solid Tumors: Results from Phase Ib
Source: Cancer Res Commun. 2023 Jul 10;3(7):1189–99. doi: 10.1158/2767-9764.CRC-22-0401 (PMC10332326; doi:10.1158/2767-9764.CRC-22-0401)
Supplement: Supplementary Table 5 — Summary of Serum Nivolumab Concentrations After the First Dose [file crc-22-0401-s06.pdf]

Supplementary Table S5. Summary of Serum Nivolumab Concentrations After the First Dose

Dosing schedule	Nivolumab dose level	E7389-LF dose level	Serum Nivolumab concentration (µg/mL)	
			C_{max}[a]	C_{min}[b]
Q2W	240 mg	1.1 mg/m^2	65.0 ± 8.91 (*n* = 7)	21.0 ± 3.97 (*n* = 6)
		1.4 mg/m^2	66.6 ± 7.20 (*n* = 6)	20.5 ± 4.50 (*n* = 5)
Q3W	360 mg	1.7 mg/m^2	114 ± 22.6 (*n* = 6)	30.1 ± 6.04 (*n* = 4)
		2.1 mg/m^2	105 ± 44.4 (*n* = 6)	25.4 ± 6.18 (*n* = 6)

Data are shown as mean ± standard deviation.

[a]Blood samples for C_{max} were collected just before the completion of infusion on C1D1; [b]blood samples for C_{min} were collected before the start of infusion of the second dose (Q2W: C1D15, Q3W: C2D1).

C_{max}, maximum serum concentration; C_{min}, minimum serum concentration; C#D#, cycle # day #; E7389-LF, eribulin liposomal formulation; Q#W, every # weeks.
