# Supplementary material for: Phase Ib/II Study of a Liposomal Formulation of Eribulin (E7389-LF) plus Nivolumab in Patients with Advanced Solid Tumors: Results from Phase Ib
Source: Cancer Res Commun. 2023 Jul 10;3(7):1189–99. doi: 10.1158/2767-9764.CRC-22-0401 (PMC10332326; doi:10.1158/2767-9764.CRC-22-0401)
Supplement: Supplementary Table 6 — Analyses of Median Changes in Plasma Protein Expression of Biomarker Analytes From Baseline to C1D8 and C1D15 in All Dosing Cohorts [file crc-22-0401-s07.pdf]

Supplementary Table S6. Analyses of Median Changes in Plasma Protein Expression of Biomarker Analytes From Baseline to C1D8 and C1D15 in All Dosing Cohorts

**Analyte**	**C1D8**			**C1D15**		
	**Median % change**	***P*-value[a]**	**Adjusted *P*-value[b]**	**Median % change**	***P*-value[a]**	**Adjusted *P*-value[b]**
6Ckine (CCL21)	−3.71	0.378	0.495	−1.92	0.814	0.830
Angiopoietin 1	−12.90	0.232	0.338	0.00	0.671	0.761
Angiopoietin 2	8.00	0.245	0.347	20.51	0.019	0.050
BAFF (B cell-activating factor)	**74.44**	<0.001	<0.001	**68.41**	<0.001	<0.001
BDNF (brain-derived neurotrophic factor)	**−71.88**	0.012	0.022	−62.11	0.116	0.203
BLC (B lymphocyte chemoattractant)	**68.00**	<0.001	<0.001	**60.00**	<0.001	<0.001
Cancer antigen 15 3	−1.19	0.782	0.816	5.56	0.129	0.206
CA9 (carbonic anhydrase-9)	**52.83**	<0.001	<0.001	0.00	0.523	0.620
Collagen IV	**31.00**	<0.001	<0.001	**16.67**	0.001	0.005
Decorin	**12.50**	0.006	0.012	−6.25	0.053	0.118
EGFR (epidermal growth factor receptor)	0.00	0.626	0.679	−3.03	0.107	0.203
Endoglin	5.17	0.784	0.816	−3.23	0.742	0.805
Fatty acid-binding protein, adipocyte	**90.32**	<0.001	<0.001	**8.33**	0.007	0.021
Factor VII	**7.35**	0.002	0.004	2.28	0.116	0.203
Hepsin	**10.70**	0.006	0.012	5.48	0.062	0.126
HER2 (human epidermal growth factor receptor 2)	4.55	0.958	0.958	9.26	0.193	0.266
HGFR (hepatocyte growth factor receptor)	0.00	0.458	0.557	1.75	0.134	0.207
ICAM1 (intercellular adhesion molecule 1)	**18.88**	<0.001	<0.001	**20.22**	<0.001	<0.001
IFNγ (interferon gamma)	**325.00**	<0.001	<0.001	**69.23**	<0.001	<0.001
IGFBP1 (insulin-like growth factor-binding protein 1)	**28.13**	0.023	0.042	26.32	0.126	0.206
IGFBP2 (insulin-like growth factor-binding protein 2)	1.45	0.504	0.598	3.57	0.187	0.264
Interleukin 12 Subunit p40	−14.29	0.525	0.606	7.23	0.125	0.206
IL13 (interleukin 13)	**42.59**	<0.001	<0.001	**142.11**	<0.001	<0.001
IL18 (interleukin 18)	**83.00**	<0.001	<0.001	**45.16**	<0.001	<0.001
IL18BP (interleukin 18 binding protein)	**33.33**	<0.001	<0.001	**30.00**	<0.001	<0.001
Interleukin 1 receptor antagonist	10.96	0.032	0.056	**15.32**	0.009	0.027
IL8 (interleukin 8)	**33.78**	<0.001	<0.001	14.29	0.062	0.126

Analyte	C1D8			C1D15		
	**Median % change**	***P*-value[a]**	**Adjusted *P*-value[b]**	**Median % change**	***P*-value[a]**	**Adjusted *P*-value[b]**
IP10 (interferon gamma-induced protein 10, CXCL10)	**77.19**	<0.001	<0.001	**34.04**	<0.001	<0.001
ITAC (interferon-inducible T-cell alpha chemoattractant, CXCL11)	**31.11**	<0.001	<0.001	**24.00**	0.002	0.006
Kallikrein 5	0.00	0.258	0.356	0.00	0.667	0.761
Kallikrein 7	−2.39	0.357	0.479	−12.60	0.022	0.057
MCP1 (monocyte chemotactic protein 1)	**38.65**	<0.001	<0.001	**−31.25**	<0.001	0.001
MIF (Macrophage migration inhibitory factor)	−17.86	0.454	0.557	−15.38	0.158	0.236
MIG (monokine induced by gamma interferon, CXCL9)	**54.97**	<0.001	<0.001	**60.75**	<0.001	<0.001
MIP1β (macrophage inflammatory protein-1 beta)	**52.52**	<0.001	<0.001	2.06	0.406	0.507
MIP3β (macrophage inflammatory protein-3 beta)	**41.94**	<0.001	<0.001	**29.44**	<0.001	<0.001
MMP3 (matrix metallopeptidase 3)	0.00	0.450	0.557	−2.82	0.837	0.837
MMP9 (matrix metallopeptidase 9)	**−28.57**	<0.001	0.002	**−41.11**	0.001	0.004
PECAM1 (platelet endothelial cell adhesion molecule 1)	−2.13	0.577	0.639	0.00	0.776	0.807
Prostasin	14.34	0.034	0.058	3.91	0.424	0.514
SCF (stem cell factor)	−7.15	0.535	0.606	−6.57	0.718	0.796
SDF1 (stromal cell-derived factor 1, CXCL12)	**10.77**	<0.001	<0.001	1.66	0.232	0.311
SPD (pulmonary surfactant-associated protein)	−7.69	0.232	0.338	0.00	0.408	0.507
TG (thyroglobulin)	**24.30**	<0.001	<0.001	14.42	0.108	0.203
TIE2 (tyrosine kinase immunoglobulin and epidermal growth factor homology domains 2, TEK)	**22.22**	<0.001	<0.001	**11.11**	<0.001	<0.001
TNC (tenascin-C)	**180.49**	<0.001	<0.001	**99.22**	<0.001	<0.001
UPAR (urokinase-type plasminogen activator receptor)	3.64	0.095	0.151	3.49	0.164	0.239

Analyte	C1D8			C1D15		
	**Median % change**	***P*-value[a]**	**Adjusted *P*-value[b]**	**Median % change**	***P*-value[a]**	**Adjusted *P*-value[b]**
VEGF (vascular endothelial growth factor)	−6.15	0.863	0.880	−9.36	0.045	0.110
VEGFD (vascular endothelial growth factor D)	−7.02	0.037	0.060	−5.73	0.270	0.353
VEGFR2 (vascular endothelial growth factor receptor 2)	3.57	0.208	0.322	−5.26	0.768	0.807
VEGFR3 (vascular endothelial growth factor receptor 3)	**16.67**	0.008	0.016	7.32	0.051	0.118

Bolded values indicate change ($P<0.05$) determined by adjusted *P*-value; pink-shaded cells represent an increase from baseline, and blue-shaded cells represent a reduction.

[a]Calculated through Wilcoxon analysis; [b]calculated through Wilcoxon analysis; and adjusted for false discovery rate by Benjamini–Hochberg procedure.

C#D#, cycle # day #; CCL, C-C motif chemokine ligand; CXCL, C-X-C motif chemokine ligand; TEK, TEK receptor tyrosine kinase.
