# Supplementary material for: Phase Ib/II Study of a Liposomal Formulation of Eribulin (E7389-LF) plus Nivolumab in Patients with Advanced Solid Tumors: Results from Phase Ib
Source: Cancer Res Commun. 2023 Jul 10;3(7):1189–99. doi: 10.1158/2767-9764.CRC-22-0401 (PMC10332326; doi:10.1158/2767-9764.CRC-22-0401)
Supplement: Supplementary Table 7 — Tumor Biomarkers: Assessment of Immune Phenotypes [file crc-22-0401-s08.pdf]

Supplementary Table S7. Tumor Biomarkers: Assessment of Immune Phenotypes

E7389-LF Dosage	Patient No.[a]	Tumor type	Best Overall Response	PD-L1 Combined Positive Score at Baseline, %	Immune Phenotype[b]	
					Screening	C2D1
1.1 mg/m² Q2W	1	Paget's disease	PD	35	Desert	-
	2	Cholangiocarcinoma	PR	0	Desert	Inflamed
	3	Sarcoma	NE	NA	NA	-
	4	Small cell lung cancer	SD	0	Desert	NA
	5	Colorectal cancer	PD	NA	Desert	-
	6	Gastric cancer	PD	5	Desert	-
	7	Gastric cancer	PD	0	Desert	-
1.4 mg/m² Q2W	8	Ovarian cancer	PD	0	Desert	Inflamed
	9	Neuroendocrine carcinoma	PD	0	Excluded	Excluded
	10	Urothelial cancer	SD	0	Excluded	Inflamed
	11	Pancreatic cancer	PD	25	Desert	-
	12	Cholangiocarcinoma	SD	0	Desert	Excluded
	13	Thymic carcinoma	SD	33	Desert	Desert
1.7 mg/m² Q3W	14	Adenoid cystic carcinoma	SD	NA	Desert	Desert
	15	Ovarian cancer	PD	0	Desert	Inflamed
	16	Colorectal cancer	PD	14	Desert	-
	17	Thymic carcinoma	PR	65	Inflamed	Inflamed
	18	Ovarian cancer	PD	6	Inflamed	-
	19	Thymic carcinoma	PR	6	Inflamed	NA
2.1 mg/m² Q3W	20	Small cell lung cancer	PR	20	Excluded	NA
	21	Neuroendocrine carcinoma	PD	0	NA	Desert
	22	Ovarian cancer	SD	40	Excluded	-
	23	Thymic carcinoma	SD	40	Excluded	Excluded
	24	Gastric cancer	SD	7	Excluded	Excluded
	25	Primary origin unknown	PD	7	Inflamed	Inflamed

Highlighted patients are those who had an immune-desert or immune-excluded phenotype at screening and an immune-inflamed phenotype at C2D1.

[a]For use to compare patient results to **Supplementary Figure S3** and **S4**; [b]inflamed = high degree of cytotoxic T cell infiltration; excluded = T cells at invasive margin of tumor, none in tumor bed; desert = T cells absent from tumor and margins. "-" represents phenotypes that could not be evaluated for certain reasons (eg no biopsy conducted). "NA" represents phenotypes where immunohistochemistry test could not be conducted (eg no tumor tissue was collected in sample).

C2D1, cycle 2 day 1; E7389-LF, eribulin liposomal formulation; NA, not available; NE, not evaluable; PD, progressive disease; PD-L1, programmed cell death ligand 1; PR, partial response; Q#W, every # week; SD, stable disease.
