# Supplementary material for: Phase Ib/II Study of a Liposomal Formulation of Eribulin (E7389-LF) plus Nivolumab in Patients with Advanced Solid Tumors: Results from Phase Ib
Source: Cancer Res Commun. 2023 Jul 10;3(7):1189–99. doi: 10.1158/2767-9764.CRC-22-0401 (PMC10332326; doi:10.1158/2767-9764.CRC-22-0401)
Supplement: Supplementary Figure 1 — S1. Box-and-Whisker Plot of Neutrophil Measurements During Cycle 1 for the (A) Q3W and (B) Q2W Dosing Groups. [file crc-22-0401-s09.pdf]

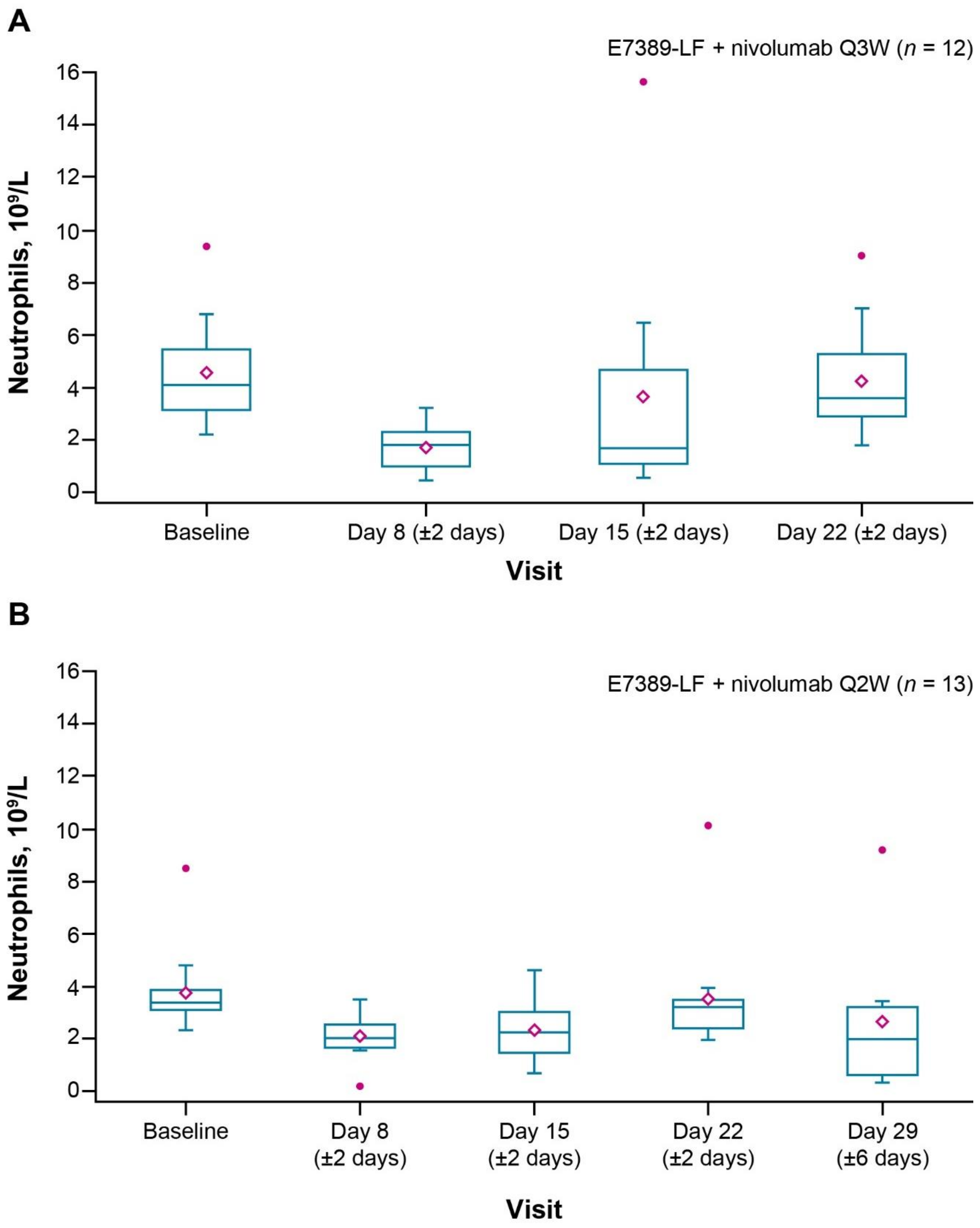

Supplementary Figure S1.

Box-and-Whisker Plot of Neutrophil Measurements During Cycle 1 for the (A) Q3W and (B) Q2W Dosing Groups. The boxes represent median and quartiles, bars represent range, diamonds ◊ represent means, and circles ○ represent outliers.

E7389-LF, eribulin liposomal formulation; Q#W, every # weeks.
