# Supplementary material for: Phase Ib/II Study of a Liposomal Formulation of Eribulin (E7389-LF) plus Nivolumab in Patients with Advanced Solid Tumors: Results from Phase Ib
Source: Cancer Res Commun. 2023 Jul 10;3(7):1189–99. doi: 10.1158/2767-9764.CRC-22-0401 (PMC10332326; doi:10.1158/2767-9764.CRC-22-0401)
Supplement: Supplementary Figure 2 — S2. Linear (A) and Semi-logarithmic (B) Plasma Concentration Profiles of Eribulin After Administration of E7389-LF. [file crc-22-0401-s10.pdf]

A

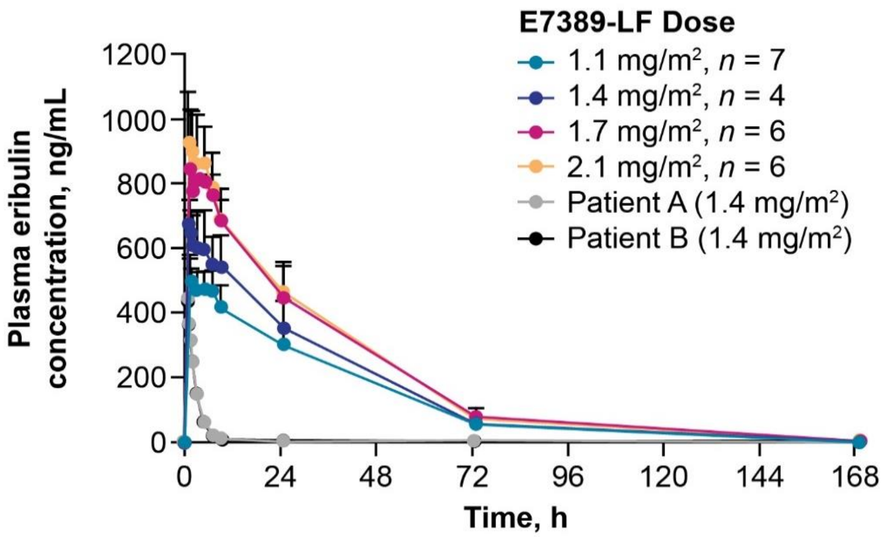

B

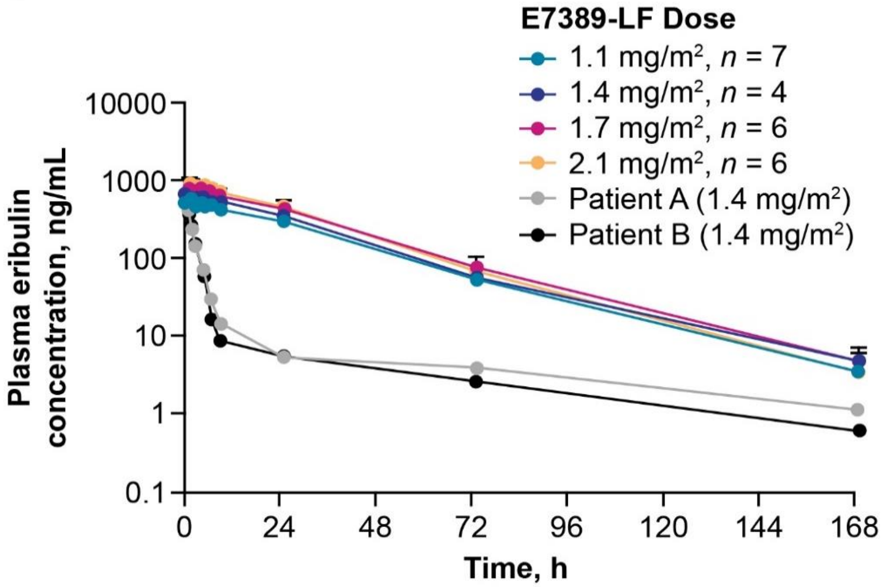

Supplementary Figure S2.

Linear (A) and Semi-logarithmic (B) Plasma Concentration Profiles of Eribulin After Administration of E7389-LF. Plasma concentrations are shown as mean + standard deviation. Two patients (represented here as Patient A and Patient B) in the E7389-LF 1.4 mg/m^2 cohort showed unique plasma eribulin concentration profiles (2-phasic decline of plasma eribulin concentration and lower AUC) after the administration of E7389-LF on cycle 1 day 1. The cause is currently unknown, but their pharmacokinetic profiles were considered to be outliers because the profiles were markedly different from those of the other patients in this study and the previous E7389-LF monotherapy study. Therefore, the 2 patients were excluded from the summary of plasma concentration profiles.

E7389-LF, eribulin liposomal formulation.
