# Supplementary material for: Phase Ib/II Study of a Liposomal Formulation of Eribulin (E7389-LF) plus Nivolumab in Patients with Advanced Solid Tumors: Results from Phase Ib
Source: Cancer Res Commun. 2023 Jul 10;3(7):1189–99. doi: 10.1158/2767-9764.CRC-22-0401 (PMC10332326; doi:10.1158/2767-9764.CRC-22-0401)
Supplement: Supplementary Figure 3 — S3. Imaging of 2 Patients with Cholangiocarcinoma During Treatment. [file crc-22-0401-s11.pdf]

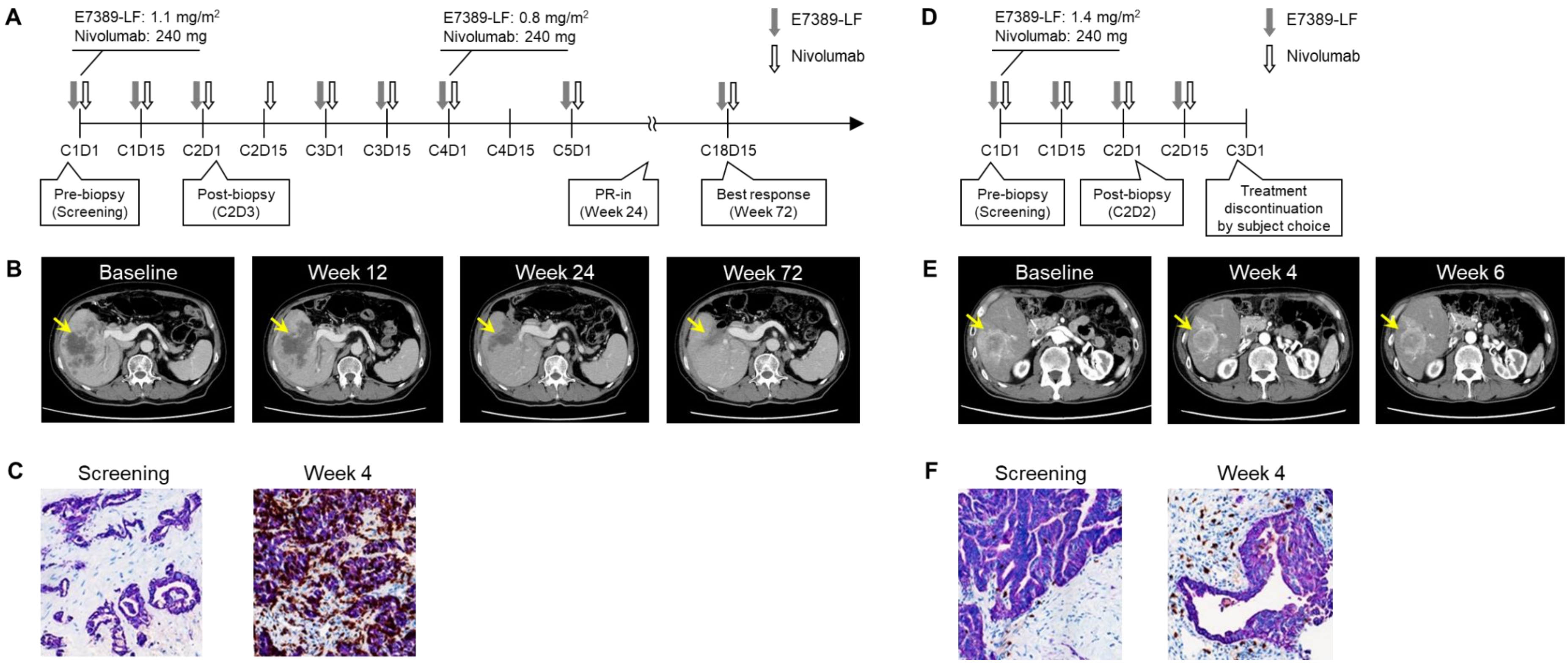

Supplementary Figure S3.

Imaging of 2 Patients with Cholangiocarcinoma During Treatment. The timeline shows the clinical course of 2 patients with cholangiocarcinoma (A, D); imaging shows the change of the liver lesion over time (B, E); IHC imaging of PanCK tumor cells (purple) and CD8 cytotoxic T cells (brown) (C, F). From baseline to C2D1, the patient in the E7389-LF 1.1 mg/m^2 Q2W cohort (Patient 2[a]; A, B, C) had their immune phenotype change from immune-desert to immune-inflamed, while the patient in the E7389-LF 1.4 mg/m^2 Q2W cohort (Patient 12[a]; D, E, F) had their phenotype change from immune-desert to immune-excluded. [a]Per **Supplementary Table S7**.

C#D#, cycle # day #; E7389-LF, eribulin liposomal formulation; IHC, immunohistochemistry; PD, progressive disease; PR, partial response; Q#W, every # weeks.
