# Supplementary material for: Phase Ib/II Study of a Liposomal Formulation of Eribulin (E7389-LF) plus Nivolumab in Patients with Advanced Solid Tumors: Results from Phase Ib
Source: Cancer Res Commun. 2023 Jul 10;3(7):1189–99. doi: 10.1158/2767-9764.CRC-22-0401 (PMC10332326; doi:10.1158/2767-9764.CRC-22-0401)
Supplement: Supplementary Figure 4 — S4. Gene Expression Profiling in Biopsy Tissues [file crc-22-0401-s12.pdf]

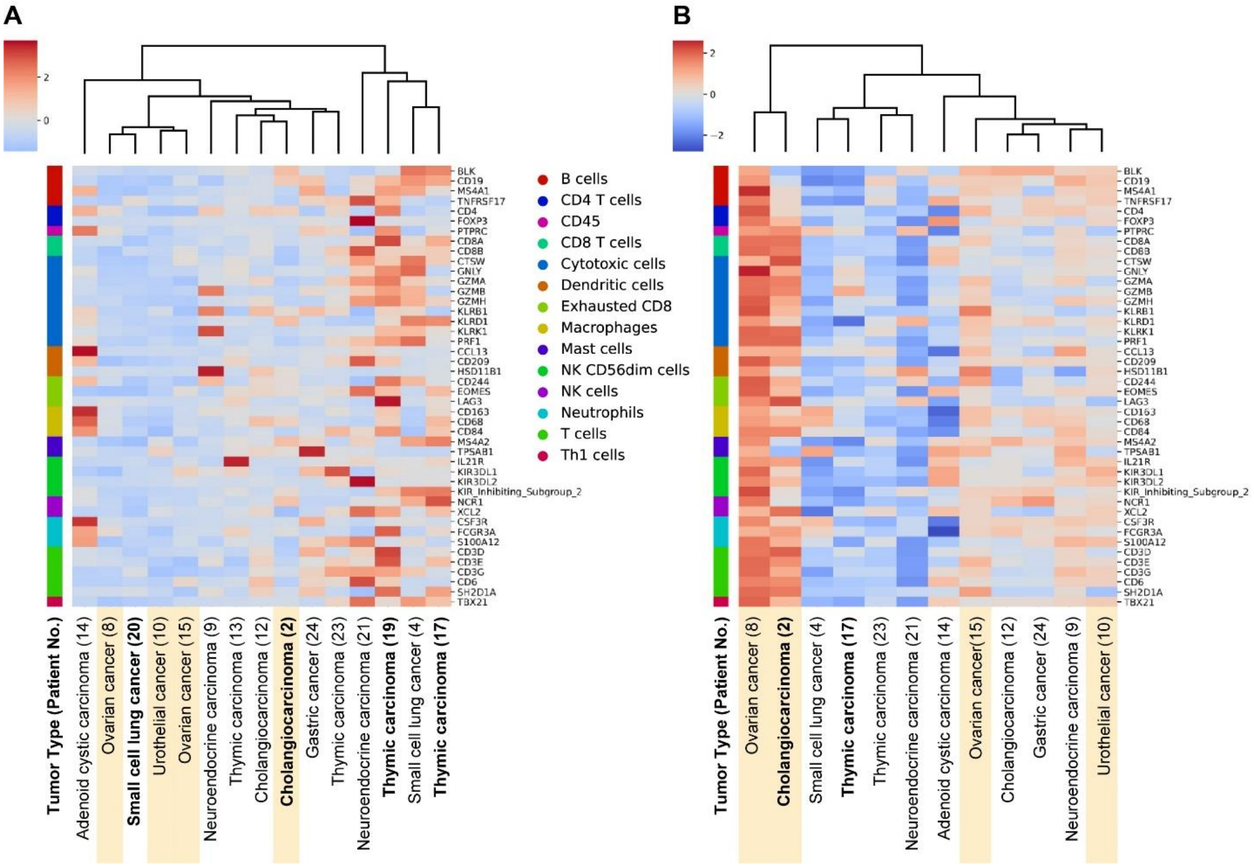

Supplementary Figure S4.

Gene Expression Profiling in Biopsy Tissues by (A) Immune Cell Type Genes at Baseline and (B) Gene Fold-Change from Baseline to Cycle 2 Day 1. Gene analysis was conducted using nCounter PanCancer Immune Profiling Panel (NanoString). Patient number as reported in **Table 4**. Bolded cells represent patients with a partial response and colored cells represent patients who changed phenotype by cycle 2 day 1. Heat maps show expression levels of cell-type-specific marker genes as raw expression values for baseline samples and Log2 values for fold changes from baseline to cycle 2 day 1. Each gene expression was normalized by z-score, red indicated high score, blue indicated low score, and patients were clustered with Ward method based on Euclidean distances.
